# Impact of a Search Engine on Clinical Decisions Under Time and System Effectiveness Constraints: Research Protocol

**DOI:** 10.2196/12803

**Published:** 2019-05-28

**Authors:** Anton van der Vegt, Guido Zuccon, Bevan Koopman, Anthony Deacon

**Affiliations:** 1 School of Information Technology and Electrical Engineering The University of Queensland St Lucia Australia; 2 Australian eHealth Research Centre, The Commonwealth Scientific and Industrial Research Organisation Brisbane Australia; 3 School of Medicine University of Queensland St Lucia Australia

**Keywords:** information storage and retrieval, clinical decision making, evidence-based medicine

## Abstract

**Background:**

Many clinical questions arise during patient encounters that clinicians are unable to answer. An evidence-based medicine approach expects that clinicians will seek and apply the best available evidence to answer clinical questions. One commonly used source of such evidence is scientific literature, such as that available through MEDLINE and PubMed. Clinicians report that 2 key reasons why they do not use search systems to answer questions is that it takes too much time and that they do not expect to find a definitive answer. So, the question remains about how effectively scientific literature search systems support time-pressured clinicians in making better clinical decisions. The results of this study are important because they can help clinicians and health care organizations to better assess their needs with respect to clinical decision support (CDS) systems and evidence sources. The results and data captured will contribute a significant data collection to inform the design of future CDS systems to better meet the needs of time-pressured, practicing clinicians.

**Objective:**

The purpose of this study is to understand the impact of using a scientific medical literature search system on clinical decision making. Furthermore, to understand the impact of realistic time pressures on clinicians, we vary the search time available to find clinical answers. Finally, we assess the impact of improvements in search system effectiveness on the same clinical decisions.

**Methods:**

In this study, 96 practicing clinicians and final year medical students are presented with 16 clinical questions which they must answer without access to any external resource. The same questions are then represented to the clinicians; however, in this part of the study, the clinicians can use a scientific literature search engine to find evidence to support their answers. The time pressures of practicing clinicians are simulated by limiting answer time to one of 3, 6, or 9 min per question. The correct answer rate is reported both before and after search to assess the impact of the search system and the time constraint. In addition, 2 search systems that use the same user interface, but which vary widely in their search effectiveness, are employed so that the impact of changes in search system effectiveness on clinical decision making can also be assessed.

**Results:**

Recruiting began for the study in June 2018. As of the April 4, 2019, there were 69 participants enrolled. The study is expected to close by May 30, 2019, with results to be published in July.

**Conclusions:**

All data collected in this study will be made available at the University of Queensland’s UQ eSpace public data repository.

**International Registered Report Identifier (IRRID):**

DERR1-10.2196/12803

## Introduction

Clinicians are routinely faced with medical questions related to their patient interactions [1]. Studies conducted with primary care physicians show that on average between 0.07 and 1.85 questions are generated per patient encounter [2], or a little under 1 question per hour [3]. Of these questions, many are often left unanswered, as demonstrated by 3 studies in the United States [3-5] where 63.76% (702/1101), 44.91% (477/1062), and 70.2% (207/295) of the medical questions raised by the clinicians were left unanswered. Clinicians are expected to seek and apply the best evidence to answer their clinical questions, according to an evidence-based-medicine approach to clinical decision making [6,7]. Search engines provide a means for clinicians to access scientific literature while on the job. However, physicians suggest that lack of time and the belief that the system will not provide a definitive answer are 2 of the primary barriers to pursuing an answer [4,5]. So, the question remains: how effective are scientific literature search engines at supporting clinicians in making better clinical decisions. This study aims to address this question.

### Study Aims

The overall aim of this study is to examine the suitability of using a search engine to search scientific literature to enable time-pressured clinicians to make better clinical decisions. To support this assessment, the following 3 research questions (RQs) will be addressed:

RQ1: Does the use of a Web-based scientific literature search system enable clinicians to make better clinical decisions?

RQ2: How does time pressure impact clinical decision quality?

RQ3: Does a significantly better search system, as measured by standard information retrieval (IR) evaluation measures, translate to better and faster clinical decisions?

### Significance of This Study

This study will inform both health care providers, with regard to system selection to suit their use case, and system designers, with regard to evidence selection and search effectiveness requirements. It will also contribute a rich data collection for future research purposes, including specifically:

- Clinician query sets to analyze the impact of query quality on clinical decision making.
- Clinician evidence sets (ie, actual text selected from the literature by clinicians to provide evidence for their clinical answers) to analyze their relationships with clinical questions, clinician queries, search retrieval snippet cues, clicked documents, and answer quality.
- Clinician document relevance ratings to analyze the relationship with snippet design, clicked documents, judged relevance, selected evidence, and answer quality.
- Clinician search engine results page (SERP) interaction, including read time and clicks to help identify patterns of search behavior and how this relates to clinical decision quality.
- Search and answer time breakdown to identify where time is spent during search for evidence.

### Sources of Evidence

IR systems can use one or more of many different sources of evidence to help clinicians answer their clinical questions, including scientific literature, best practice information, guidelines, or synthesized information, such as that generated by UpToDate (Wolters Kluwer) [8]. Haynes identified the *5S* levels of organization of evidence from health care research [9], which depicts a pyramid of health care evidence with journal studies at the base followed by syntheses, synopses, summaries, and finally systems, such as computerized decision support systems, at the top. Of interest to this research is the use of scientific medical literature (SML), such as that found in the MEDLINE (US National Library of Medicine) [10] database or accessed via PubMed (US National Library of Medicine). It includes original research and meta studies, such as systematic reviews, and is represented by the bottom 2 layers of Haynes’ pyramid of evidence. SML is widely used across the medical research community and the public [11], but it is also a common source of evidence used by clinicians [12,13] to support their clinical queries.

### Physician Preference Versus Suitability?

Although SML is used widely by clinicians, larger studies conducted across medical institutions suggest that it is not a preferred source of evidence for busy clinicians. In particular, Ellsworth et al [14] found in a survey of 450 clinicians across the Mayo Clinic that 56.8% (255/450) of respondents preferred synthesized information sources versus 12.9% (58/450) who preferred original research. Hoogendam et al [15] studied the clinical evidence preferences of 70 clinicians in a Dutch academic medical center over the course of 18 months. Their study found that while answering 1305 patient-related questions, clinicians chose to use UpToDate 78.49% (883/1125) of the time rather than PubMed. Hoogendam et al asserted that the time required to find an answer was the most likely explanation for this bias, noting that clinicians spent, on average, less than 5 min pursuing a question.

Clinician preference for synthesized evidence, rather than SML, is at best an implicit indicator of the suitability of SML search systems for their clinical needs. However, the time clinicians have available for answering their questions, and therefore the time needed to search for a definitive answer, is likely to be an important facet of SML system suitability to be incorporated within our study.

### Previous Search System Studies

It is difficult to find conclusive evidence supporting SML as the sole source of evidence for clinicians under strict time constraints. Dunn et al [12] analyzed surveys from 14,544 clinicians examining the impact of evidence search on patient care. They found that 75.33% (10,956/14,544) of respondents used more than one evidence source and that journals (print and Web) and MEDLINE were the top 2 sources used. They concluded that these sources are an effective component in providing clinical answers; however, the use of UpToDate and other evidence sources made it difficult to evaluate MEDLINE in a stand-alone context. McKibbon et al [13] assessed the successful answering of 46 clinical questions across 23 clinicians. The physicians took on average 13 min to answer each question and could reference multiple data sources of their choice, including PubMed and MEDLINE; however, their correct answer rate only improved from 18 (39% [18/46]) questions correct presearch to 19 (41% [19/46]) correct after using a search system. Westbrook et al [16], on the contrary, found more extensive improvement with the use of a search system. They studied the answer accuracy of 8 clinical questions presented to 75 clinicians, including nurses and doctors. The study was designed to simulate realistic time pressures, so participants were given 10 min per question; however, this limit was not enforced. Although participants completed the 8 questions within 80 min, it was unclear whether some questions took longer than 10 min to complete. The study showed that the introduction of a clinical evidence search system improved the correct answer rate from 174 (29.0% [124/600]) correct questions without the system to 298 (49.7% [298/600]) correct with the system. The search system comprised 6 sources of evidence, PubMed included.

These studies show that an evidence search system can be effective to help clinicians make better clinical decisions and that SML may be a helpful component of a broader range of evidence sources; however, they do not confirm whether an SML search system is suitable as a stand-alone system for the same task. Studies conducted where SML was the sole source of evidence include the ones by Hersh et al [17,18]. In their first study, 19 medical students and 8 nursing students answered 3 medical questions each [17]. The correct answer rate improved from 39 (45% [39/87]) correct answers to 66 (76% [66/87]) after searching MEDLINE alone. This is a much higher increase than found in the study by Westbrook et al; perhaps attributable to the questions asked, some of which were examination style, and the 1-hour timeframe to complete the questions. In the second study, 45 medical and 21 nurse practitioner students answered a total of 324 questions [18]. The use of MEDLINE-only search improved correctness from 104 (32.1% [104/324]) correct to 150 (46.3% [150/324]) overall; however, the nursing students showed a small improvement of just 3 percentage points. These studies [17,18] do focus on SML alone; however, the longer allowable answer timeframes and the conflicting results motivate the authors of this study to more tightly control the user study, similar to Westbrook et al, but with enforced time limits and a single evidence source.

### Time Constraints and Time Pressure

According to Ordonez and Benson [19], time constraints exist whenever there is a deadline for a task; however, for the task performer to be time pressured, the time constraint must induce stress such that they feel the need to cope with the limited time. In our study, time pressure will be induced by specifying to the participant, and enforcing, a time limit for searching the SML for an answer.

In the field of psychology, experiments have revealed many coping mechanisms that impact the task performer’s decisions [20-23]. Many of these coping mechanisms are relevant to clinical decision making, for example, Wright [23] found that under significant time pressure, subjects changed their decision-making strategy, used fewer information attributes to make their decision and were more reliant on negative attributes, that is, those that had negative consequences. In Edland and Svenson’s review of the literature of time-pressured decision making [21], they noted that time pressure can lead to a shallower search for information across alternatives. Svenson and Benson found that task performers will also change their decision strategy when put under time pressure [20].

Some of these behaviors have been explored in the IR field. Chang and Wei explored the impact of time constraints on users’ search strategy [24] and found significant differences between users with or without a time constraint: users under time constraints tended to view less documents and spend more time on the search engine results page. Crescenzi et al [25,26] confirmed that searchers under time-constrained conditions reported significantly greater time pressure, felt that the tasks were more difficult, and felt less satisfied with their performance. This outcome prompts the question of whether or not this lower satisfaction in performance correlates to poorer decisions. The influence of time pressure within the clinical setting has been studied by Tsiga et al [27]. In their study of 34 general practitioners, practicing within a town in Greece. They found that under time pressure, clinicians asked less questions regarding symptoms and conducted less thorough physical examinations for a given clinical scenario. This study will examine the impact of time pressure on clinical decisions. Time pressure is a major barrier to using an evidence search system [4,5], and the time-consuming nature of using an SML system [28], such as PubMed, may suggest it is inappropriate under certain time constraints. By varying the time available to search for evidence, this study will explore the relationship between the time a clinician has available to search for answers and the quality of their clinical decisions.

### Search System Effectiveness

A less obvious factor that may also impact the suitability of SML search systems for time-pressured clinicians is the effectiveness of the search engine. Intuitively, a more effective system that provides more relevant literature for the clinician’s question is more likely to speed up the answer process and, therefore, present SML as a more suitable evidence source. Studies conducted outside of health have shown that search system effectiveness can impact user search behavior, performance, and satisfaction [29-32]. In particular, Allan et al [31] varied the system effectiveness, as measured by binary preference, and captured the time it took participants to find answer facets to specific questions. They found that for specific bands of improved system effectiveness, user performance also improved, including reduced time on task, less errors, and an increased rate of finding new, correct answers. This is in contrast with the study by Turpin et al [29] who found no significant relationship between system effectiveness, as measured by mean average precision (MAP) and user performance for a simple precision-based task and only a weak relationship for a simple recall-based task.

System effectiveness was implicitly excluded in the health domain studies above by using the same search system throughout each study [17,18,33]. To our knowledge, our study will be the first to research the impact of search system effectiveness on clinician decision making.

In summary, the aim of this study is to examine the suitability of using a search engine to search scientific literature to enable time-pressured clinicians to make better clinical decisions. The impact of both time pressure and search system quality on clinical decision making will be assessed.

## Methods

### Study Design

**Figure 1:**
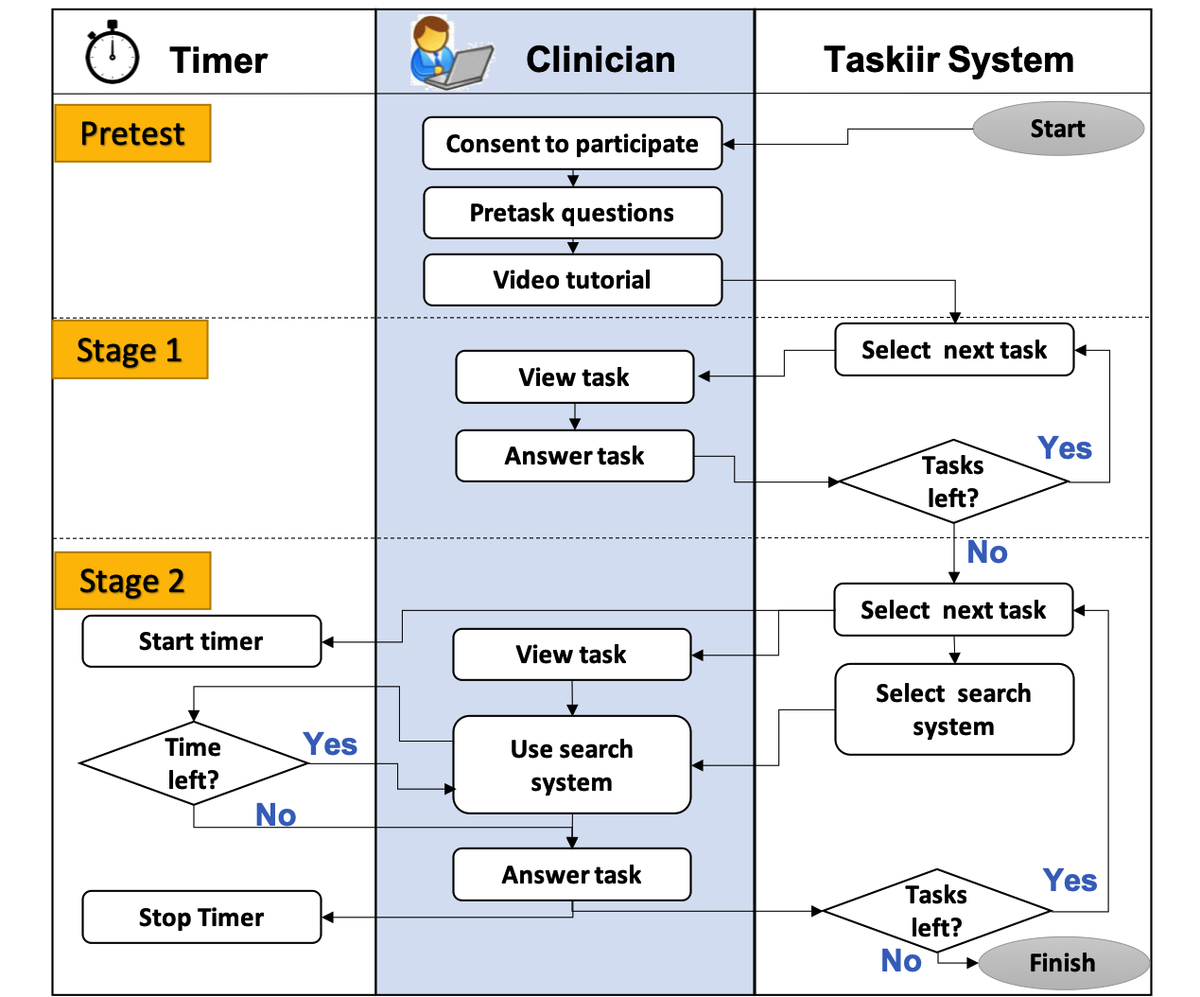
Process flow diagram of study shows both stages of the study. Stage 1 is untimed and the clinician has no access to any support resource. In stage 2, each question is timed and the participant is allocated a search engine to use for each question. If time runs out, the participant is brought directly to the task completion page.

A total of 96 participants consisting of practicing clinicians and final year medical students are provided with 16 clinical scenarios, each with a single question. Figure 1 depicts the study steps. The participants must firstly answer the questions without any supporting evidence. In the second stage of the study, the same set of clinicians are provided with the same 16 clinical scenarios and an SML search system. A bespoke best-match SML search system, called Taskiir, was used to avoid any experience variation from using the well-known PubMed interface, as described by Yoo and Mosa [34]. The participants will be constrained to one of 3, 6, or 9 min to search for suitable evidence and complete the task. The time allocated to each user for each task will change depending on the timing cohort they are assigned to (see Task Order and System Rotation section for details). In total, 2 SML search systems with the same user interface, but with significantly different search performance, will be provided to the participants for alternating questions. In this way, the presearch and postsearch correct answer rate by participant and by system will be captured.

To enable comparison with previous studies, much of the method employed by Westbrook et al [33] is replicated, including the use of 6 of the 8 clinical questions used in that study. The main differences with the Westbrook et al study are (1) the varied and strict time limits set to search and answer each question; (2) the use of medical literature only for evidence, rather than the 6 sources they used; and (3) the use of 2 search systems with different search performance.

### Participants

A convenience sample of 96 practicing clinicians and final year medical students, including nurses, general practitioners, and hospital physicians, will be asked to participate. The practicing clinical participants must be Australian registered clinicians residing in Australia. All participants must have access to a computer with an internet connection. Participants will be offered a small honorarium (Aus $50 gift card) to complete the assessment and will be recruited via mail, email, and Web-based noticeboards directed to medical student societies, clinical departments in hospitals, public health area networks, and medical faculties at Australian universities.

### Procedures

Participants will be asked to complete a 2-hour, Web-based assessment of a medical SML search system called Taskiir. After voluntary consent is received, the participants are allocated their login details via email. In the email, the participant is advised that they can perform the study in multiple sittings, within a 2-week period, at a time to suit them and that they must use their laptop/computer (not iPad) to access the study on the Web. They were also encouraged to ask for help, via email, if they had any queries or problems. After testing the system with clinicians, we found that trying to complete all 16 questions in a single sitting was too onerous for some people, either because they did not have 2-hour time blocks available or they found the workload too mentally fatiguing. The system was reconfigured so that after completing any task, the participant could stop and resume again at the next task. All such pauses were recorded by the system.

#### After Initial Login

The participant is asked 7 questions to capture demographic data, search, and medical experience (see Multimedia Appendix 1) as well as sleep information. A 5- to 10-min video tutorial follows where the study is described in more detail and the participant is shown how to use the SML search engine. The tutorial emphasizes that the participant must answer the question without the aid of other people or by looking at other resources. Once complete, the participant is shown specific instructions (provided in Multimedia Appendix 2 that again reinforce the participant’s obligation to perform the test alone, before they are permitted to move onto the 2-stage assessment.

#### In Stage 1

A total of 16 clinical tasks are presented to the participant, one at a time. To complete each task, the clinician must answer a single question within a few minutes, although this time limit is not enforced. In addition, 14 of the 16 tasks require the participant to select 1 of 4 answers (yes, no, conflicting evidence, and do not know) and the other tasks require a 1- to 2-word answer. At the end of the last task, the system will move the participant to stage 2 of the study.

#### In Stage 2

The participant must complete the same 16 tasks in the same order as stage 1; however, the participant must now use Taskiir to help them answer the question and to find evidence to support their answer. Evidence is collected by the participant selecting text, images, or both from the source documents they read. The time allocated to search for each task is set according to the timing cohort the participant belongs to and will be one of 3, 6, or 9 min. The participant is told of the time allocation at the start of each question and a minute-by-minute countdown timer is always visible to the participant; warnings are given 30 seconds before time-out. At time-out, the screen is blocked, and the participant is taken to the task completion screen to enter their final details. Other methods of communicating the time limit were trialed during development of the system. In the end, the above method was chosen because it provided a balance between (1) making the participant aware of the time allocated for each question, (2) avoiding time anchoring (where the participant incorrectly assumed all questions are allocated the same time as the first question), (3) keeping them updated with the time remaining so they do not run out of time without warning, and (4) not distracting the participant with time information (eg, using a second-by-second countdown timer that diverted too much attention away from the task).

As this is the first such study measuring the impact of time variation, a few time limits covering a wide range are required to generate significant differences in the outcomes. A useful starting point to establish these time limits is the average completion time of 6.1 min per question, reported in the Westbrook study [33]. From the same study, the SD, based on the average completion times for each of the 8 questions, across 2 systems, is 3.1 min. Therefore, time limits are set at the average question answer time (6 min) and approximately 1 SD either side of this (3 and 9 min). These limits should induce time pressures for 84% of questions with a 3-min time limit, 50% of questions with a 6-min time limit, and 16% of questions with a 9-min time limit. From previous studies, realistic answer timeframes for busy clinicians should be below 5 min [3,15,35], so the 3 proposed time limit cohorts will encompass this pragmatic indicator of search time suitability.

The timer is stopped during the system search for documents to eliminate the system search time variation or other network/system delays that may bias the overall search time available. System search time starts when the participant clicks the search button and ends when the screen is populated with the search results and is available for use. The question timer will be stopped for each search conducted, including a *Move Next* or *Move Previous* on the search screen. Participants will be told that search time is excluded from the timing to alleviate any additional time stress they may feel because of a perceived or actual slow system.

A control group of participants that could use the information system without time constraints was considered; However, it was decided that numerous similar previous studies, such as that of Westbrook [16], had already generated results that could be compared with the outcomes of this study. Allocating test participants to a control group without time constraints would reduce the statistical power of any test results achieved here and expanding the participant set was not feasible for time and cost reasons.

#### Data Capture

Immediately after initial login, participant information is captured as per the table in the Multimedia Appendix 1. Data capture then occurs on both the presearch and postsearch answer screens, as listed in Table 1. All system interactions will also be captured including (1) overall time spent searching for and answering each question; (2) dwell times before first query, on the SERP screen, on the document viewing screen, and on the answer screen; (3) the participant’s search query terms and resulting SERP; (4) documents selected from the SERP; (5) evidence selected by the participant from the documents they are viewing; and (6) relevance ratings by the participant of the documents they view (essential, helpful, duplicate-essential, duplicate-helpful, and not helpful). Multimedia Appendix 3 itemizes the search interaction times and how these relate to the study timings. Although desirable, it is not possible to question the participant regarding the utility of each search system because the user is not made aware of which search system is in use for each task.

Anonymity of the data collected is maintained by (1) identifying users and all of their interactions with a random user identification within the data capture system, (2) having no participant identification information stored in the same system database, and (3) capturing only generic participant information (see Multimedia Appendix 1) that could not be used to identify an individual.

### Availability of Data and Material

The datasets generated and/or analyzed during this study as well as access to the software for the Taskiir search system are currently not publicly available because the study is still underway and therefore not complete.

At the completion of the study, the following research data will be made available on the University of Queensland’s publicly accessible eSpace data repository [36]:

1. Excel spreadsheet containing all data reported by the user (as specified in the *Data Capture* section above) by task including answers to all study questions and task responses. Overall task timings will also be provided here.
2. mySQL database (anonymized) containing the raw data captured, including detailed user-task interaction timings, search terms, SERP results, SERP clicks, document selections, document relevance selections, and evidence selection text.
3. Auxiliary Excel/text files containing summarized subsets of (2), as required for further research and analysis.

### Clinical Tasks

The criteria for task selection was that each task must (1) have answers able to be found in the literature, (2) be able to be answered with yes/no/conflicting information or a single-term/phrase response, (3) be credible to a practicing clinician, and (4) have nonobvious answers. Overall, 6 of the 16 clinical questions are those produced and used by Westbrook et al [16] and are reproduced here in Table 2. The tasks consist of real-life scenarios and a clinical question for each scenario. Westbrook et al derived the tasks using clinical experts and designed them to be clinically relevant and of mixed complexity. In addition, 4 questions are sourced from Hersh et al [18], which are also clinical questions and used for the same purposes as this study. Overall, 3 questions are modified from the text retrieval conference (TREC) 2015, clinical decision support (CDS) topic set [37]. These questions were provided with diagnoses, which our medical physician (DA, MBBS), modified into a question of a similar format to the other questions. Finally, our medical physician also devised a further 3 other clinical questions for the purposes of this test. Moreover, 2 general practitioners trialed all questions for suitability.

**Table 1:** Data capture on the presearch and postsearch answer screens.

No	Data: purpose	Measurement	Presearch	Postsearch
1	Answer: Decision quality	Select (Yes/no/conflicting evidence/do not know) or type answer depending on the question	Yes	Yes
2	Confidence in answer: impact of the system on answer confidence	How confident are you in your answer? (1=no confidence, 2=a little confident, 3=moderately confident, 4=very confident, and 5=certain)	Yes	Yes
3	Perceived difficulty: relationship with time constraints and answer quality	How would you rate the difficulty of this clinical question? (presearch) and How would you rate the difficulty of the search for evidence for this task? (postsearch). (1=very easy, 2=easy, 3=neither easy nor difficult, 4=difficult, and 5=very difficult)	Yes	Yes
4	Perceived impact of time constraint on decision: relationship with decision quality and confidence	How would you rate the time you had available to make your decision? (1=not nearly enough time, 2=nearly enough time, 3=just enough time, 4=more than enough time, and 5=much more than enough time)	N/A	Yes
5	Perceived impact of time constraint on decision: relationship with decision quality and confidence	How would you rate the time you had available to collect evidence? (1=not nearly enough time, 2=nearly enough time, 3=just enough time, 4=more than enough time, and 5=much more than enough time)	N/A	Yes
6	Perceived impact of time constraint on participant’s stress level: relationship with decision quality and confidence	How much stress did you feel due to time pressure? (1=none, 2=a little, 3=a moderate amount, 4=a lot, and 5=more than a lot)	N/A	Yes

**Table 2:** Task specifications including the full task scenario supplied to the participant as well as the relevant reference from the corpus that supports the answer.

Question	Source
Cytobrush Pap Smear: Is the Cytobrush superior to a spatula for obtaining cells for Pap smears, in terms of technical quality (eg, percentage of interpretable smears)? [18]	[38,39]^a^
Glue Ear: A mother brings her 15-month-old son who has been seen three times in the past year for glue ear. She has heard that this can lead to learning and developmental problems and thinks her child may need surgery. His hearing is normal. Does current evidence support the need for the insertion of tympanostomy tubes to avoid developmental problems in this child? [16]	[40]
Asthma Inhaler: What is the best delivery device for effective administration of inhaled medication to a 5-year-old child during a moderately severe acute asthma attack? [16]	[41]
Nicotine Replacement Therapy after heart attack: A patient staying in hospital had a myocardial infarction two days ago and is now threatening to sign himself out. You suspect this is due to nicotine withdrawal. The patient wishes to stop smoking and seeks your advice on whether he can start nicotine replacement therapy. Is nicotine replacement therapy appropriate for this patient? [16]	[42,43]
Glucosamine sulfate: A 58-year-old woman with long-standing pain of osteoarthritis in knees, hips, and hands asks about the benefits of glucosamine sulfate. Does existing evidence demonstrate that glucosamine has a disease modifying role in osteoarthritis? [16]	[44]
Brown snake: A man is bitten by a brown snake and is taken to the hospital emergency department. There is clear evidence of envenoming (poisonous effects of venom). The hospital has run out of brown snake antivenom, so the patient must be given polyvalent snake antivenom (which contains antivenom for all Australian snakes). Should epinephrine be given with the antivenom to prevent anaphylaxis? [16]	[45,46]
Osteomyelitis diabetic foot: What anaerobic microorganism is most commonly found in osteomyelitis associated with diabetic foot? [16]	[47]^a^
Ultrasound for Deep Vein Thrombosis (DVT): Is ultrasound the best diagnostic test available to exclude the presence of lower extremity deep vein thrombosis? [18]	[48,49]^a^
Protein-losing nephropathy: Does dietary protein effect the level of proteinuria in patients with diabetic (a type of protein-losing) nephropathy? [18]	[50,51]^a^
Bladder Cancer: Is there evidence of an association between petroleum product exposure and bladder cancer? [18]	[52]^a^
Loin pain: A 48-year-old man presents with severe right sided loin pain and is diagnosed with a 4 mm distal ureteric calculus. Has Tamsulosin been shown to increase the chances of the calculus passing?^b^	[53,54]^a^
Breast cancer: Is oestrogen receptor positivity a better prognostic factor than human epidermal growth factor receptor 2 (HER2) overexpression for patients with breast cancer?^b^	[55]^a^
Dementia: Are the clinical effects of Mematine, when used as a sole agent in the treatment of Alzheimer’s Dementia, greatest in the “mild” stage of the disease?^b^	[56]^a^
Paroxysmal nocturnal hemoglobinuria: Is flow cytometry the most accepted laboratory investigation to confirm a suspected diagnosis of Paroxysmal Nocturnal Hemoglobinuria? [AD modified TREC CDS 2015 [37], Q14].	[57,58]^a^
Anaemia: Is the efficacy and side effect profile of oral iron polymaltose and oral ferrous sulfate equivalent when used for the treatment of iron deficiency anaemia among children? [AD modified TREC CDS 2015 [37], Q27].	[59]^a^

^a^Answer provided by author, Dr AD (MBBS).

^b^Question derived by author, Dr AD (MBBS).

In Westbrook et al’s study, 6 sources of evidence were available to search by the clinicians; however, only medical literature was provided in this study, as this was the source of evidence under investigation. To ensure that at least 1 relevant document existed in the corpus for each task, our medical physician searched through the corpus, using the search system, to identify 1 or more relevant documents. The resulting relevant PubMed sources are listed for each question in Table 2. The answers are excluded in this protocol to avoid any chance of participants viewing the answers before completing the study. However, they will be provided together with the results data.

### Corpus

The clinical information corpus used is the TREC 2014 and 2015 document collection [37,60]. This consists of a snapshot of the Open Access Subset of PubMed Central taken on January 21, 2014. It contains a total of 733,138 articles. The corpus was preprocessed according to the method employed by [61], including the removal of all HTML/XML tags, all numbers and all nonalphabetical characters. The corpus was then indexed with Galago (the Lemur Project) [62] version 3.12 using a Porter stemmer and stop words removal. After indexing, all very rare terms were also removed, that is, all terms with 3 or less occurrences in the corpus.

### Custom Search System

**Figure 2:**
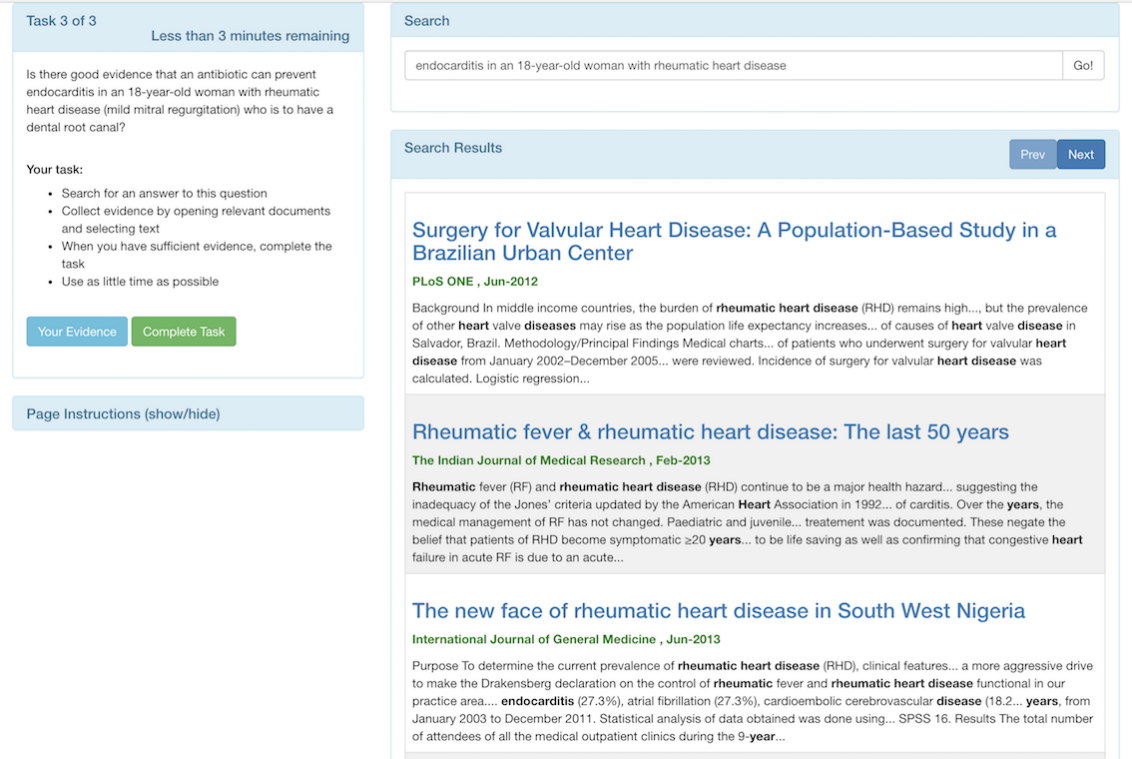
Screenshot of the Taskiir custom search system interface. Shows the task in the top left, search query box in the top right, and search results below.

A custom search engine and interface, together called Taskiir, is employed for the evidence search process (see Figure 2). Similar to normal commercial search engines, Taskiir allows the participant to write their query and perform a best match search of documents in the corpus. A snippet, highlighting matching query terms, is then provided in the SERP, which shows up below the query. Users can then select documents of interest to view the full text. While viewing the full text document, the participant can also select (with their mouse) any text or graphics that they want to use as evidence for their final answer. The participant can view their evidence or complete the task at any time. Instructions on using the system are provided on each page, and a mandatory walk-through tutorial is provided before starting the study.

To investigate the impact of search system effectiveness on clinical decision making (RQ2), Taskiir utilizes 2 search algorithms: (1) A state-of-the-art system, which is an improved version of the TREC 2015 CDS Task A winning system [61]. The TREC 2015 CDS track was targeted to identify the state-of-the-art IR system because the topics in Task A were of a similar clinical nature to the Westbrook tasks and the search corpus was the same as that used in this study. The 2 improvements made over the winning system include the removal of negated Unified Medical Language System (UMLS) terms from the UMLS query expansion terms as well as a change to the pseudorelevance feedback term weighting (from 0.75 to 0.5). All improvements resulted from tuning parameters on the CDS 2014 test collection and testing on the 2015 collection to avoid data overfitting. (2) A baseline document retrieval system consisting of a BM25 algorithm, which is a widely adopted best-match retrieval method. It is the default, out-of-the-box method employed by many search engines, including the very popular Elasticsearch (Elasticsearch BV) [63] and Lucene (Apache) systems [64]. The parameters were set to default values (K=1.2; b=0.75).

### Information Retrieval Evaluation Measures

Document retrieval performance figures for both systems are shown in Table 3. The measures depicted were the standard set chosen for the TREC 2014 and 2015 CDS task. IR system performance measures are usually calculated for a ranked retrieval of 1000 documents MAP, for example, is the average of all precision values taken at each rank where a relevant document is found. Precision at a given rank is the number of relevant documents found up to that rank divided by the rank. MAP is useful because it provides a single measurement of system performance across all queries. However, because MAP is only averaged across relevant rank positions, results can be biased toward a system retrieving fewer relevant documents but at lower rank positions. Precision at rank position 10 is simply the precision calculated at rank position 10. It is useful to identify high-precision systems that provide many relevant documents in the first 10 documents retrieved. This is often pertinent to a clinical search where clinicians have little time to view many documents. R-precision (R-prec) is the ratio r/R where r is the number of relevant documents retrieved by the system up to ranking R and R is the number of judged relevant documents for that query. Unlike MAP, R-prec takes into consideration the number of relevant documents that could be found and, therefore, is helpful for search tasks where recall is important. R-prec is a useful measure for systems that need to return many or all of the relevant documents, for example, in clinical cases that require physicians to seek alternatives, say for treatments. Discounted cumulative gain (DCG) sums the gain at each rank position (ie, the relevance grading value) multiplied by a discount factor that takes into consideration that lower ranked documents are less likely to be read. Normalized discounted cumulative gain (nDCG) compares the DCG with an ideal DCG for each rank, so that scores are normalized between 0 and 1. nDCG is designed to promote systems that provide more relevant documents higher up in the ranking.

One problem with all these standard measures is the underlying assumption that all relevant documents within the test collection are identified for each query. This is rarely the case because of cost limitations. In the measures above, unjudged documents are considered as nonrelevant; however, this may not be the case. To account for unjudged documents, Aslam et al derived 2 new measures, inferred nDCG and inferred average precision, which have become accepted methods of evaluating system retrieval performance when relevance judgements are incomplete [65].

### Sample Size

The 2 largest and most similar studies [16,18], both commenced with a presearch correct answer rate of around 30% (29% and 32%) and a postsearch rate around 50% (50% and 46%). Using this as our basis, we wanted to be able to discriminate between the postsearch correct answer rate between each of the 3 time-constrained cohorts. Therefore, to derive the sample size, we estimated that the correct answer rates might vary evenly by 10 percentage points between each group, starting at no improvement. This creates 3 datasets with average correct answer rates of 30%, 40%, and 50% for the 3-, 6- and 9-min cohorts, respectively. Applying a 2-proportion statistical comparison (ie, a 2-sample, 2-sided equality) [66], between each pair of answer rates and setting statistical power to 90%, error rate to 5%, and equal sample sizes per cohort, the minimum sample size required is 514 per cohort, which equates to 32 people per cohort sitting 16 tasks or 96 people in total.

**Table 3:** Comparison of document retrieval performance figures, across the text retrieval conference (TREC) 2015 test collection, for systems used in this study and the winning TREC 2015 system.

System	Inferred normalized discounted cumulative gain	Inferred average precision	Precision at rank position 10	R-precision	Mean average precision
Wayne State University (WSU) system^a^	0.2928	0.0777	0.4633	0.2329	0.1851
State-of-the-art system	0.3159	0.0849	0.4800	0.2401	0.1930
BM25 system	0.2168	0.0461	0.3600	0.1717	0.1114
State-of-the-art versus BM25 (%)	+46^b^ (*P*=1.2E-05)	+84^b^ (*P*=0.0056)	+33^b^ (*P*=4.1E-04)	+40^b^ (*P*=4.3E-05)	+73^b^ (*P*=7.9E-05)

^a^As per TREC 2015 CDS, task A, automatic runs listed in [37] for task summary.

^b^Significance using paired *t* test

### Task Order, System Rotation, and Task Timing

Task order and system rotation is set as per the table in Multimedia Appendix 4 for each participant to minimize confounding factors. The design is as follows:

1. A Latin square experimental design is constructed for 16 tasks and 16 participants to minimize the impact of user fatigue on specific tasks.
2. To minimize task order effects, each column of the square is randomized.
3. To incorporate a within-subject design across the system variable, 2 sets of the Latin square derived in (2) are required with alternating use of systems. The first tranche of 16 participants will start their first task with the state-of-the-art system, whereas the second tranche will start with BM25 system. In this way, across the 32 participants, each system will be used equally across all tasks and will experience the same task-order pattern.

The search time allowed for each task is controlled by applying a time limit for each task the participant performs. Participants are randomly assigned to 1 of 3 timing cohorts. The time constraint by task number is specified for each cohort in Multimedia Appendix 5. The rotation of task timing ensures that:

1. the maximum duration for search in stage 2 is fixed to 96 min for all participants
2. each task is conducted under all time constraints an equal number of times (32 per cohort)
3. a within-subject design across the time constraint variable such that each participant performs 4 to 6 tasks per time constraint
4. task time constraints are applied in the same random order according to the task order rotation Latin square, specified above

### Statistical Analyses

To assess the impact of introducing the SML search system on clinical decision quality (RQ-1), each participant’s answer, both presearch and postsearch, will be coded to right (R) or wrong by comparing the participant’s answer with an expert judged assessment (gold answer) of each task. Samples for which (1) no evidence is captured and (2) no relevant documents are marked (either as essential or helpful), by the participant for their postsearch answer, will be discarded, as the value of the search system cannot be confirmed in these cases. Therefore, the decision quality is defined by the correct answer rate (number of right answers/total sample count (N)). A further detailed analysis will be performed of the collected evidence to identify tasks where the literature may contradict the gold answers. Where this occurs, the task answers will be reviewed by experts and overall correct rates adjusted.

To assess the significance of any change in the proportion of right or wrong answers, the McNemar test will be employed because it is a nonparametric test suited to a binary result, with samples taken at 2 points in time. Nonparametric is a better model to assume, given that the data distribution is unlikely to be regular because of the different medical groupings of participants. The sign test, which is also a nonparametric test, will be used to identify any significant changes to the correct rate. To assess any differences between the participant groups (nurses, doctors, and students), a Chi-square analysis will be performed. The participant’s confidence in their answers will be assessed presearch and postsearch to identify any significant changes relating to search intervention, also using chi-square analysis.

To assess the impact of time constraints on clinical decision quality (RQ-2), the analyses above will be repeated with a breakdown by time constraint category, that is, 3, 6 and 9 min. In addition, an analysis of time-outs by constraint category will be conducted to assess the impact of time constraints on task completion. Time-outs are defined as samples where at the postsearch answer stage (1) the task timer reaches the constraint duration and (2) the participant provides no evidence to support their answer. It is assumed that in a time-out scenario, the participant was unable to complete the task. Significant differences by time-constraint category will be analyzed using the chi-square analysis. An analysis of variance (ANOVA) will be performed across confidence, difficulty, participant-perceived time impact assessments (impact of time on answer, evidence capture, and stress), and search behaviors, such as SERP dwell time, number of queries issued, number of documents opened, and the quantity of evidence items selected. To gain an understanding of the impact of providing a time constraint on the decision-making process, both the average time to search and the average proportion of available search time used will be evaluated and compared for the different task-timing samples. Tombros et al [67] used this proportional figure as a further gauge of participant stress and it can be compared with the reported stress by the participants.

To assess the impact of search engine performance on clinical decision quality (RQ-3), a similar set of analyses will be performed as that for RQ-1, except broken down by search system (state-of-the-art and BM25). In addition, the same ANOVA methods employed for time-constraint categories in RQ-2 analysis will be performed. In addition, an ANOVA will be performed across system categories and system time constraints to identify cases where system performance effects may matter most. The impact of search engine performance on clinical decision time (RQ-3) will also be assessed by evaluating the postsearch task completion times for those tasks that were completed (ie, relevant documents and/or evidence identified). This is measured in 2 ways: (1) search time only and (2) search time plus time spent filling in the answer form. Differences in search times between the systems will be assessed using the chi-square analysis. Finally, a participant-derived performance assessment of the 2 systems can be constructed by building a graded query relevance (QREL) listing (standard format for representing relevance assessments in IR), by query, based on all participants’ relevance ratings. Using this QREL, a recomparison of the 2 systems can be evaluated and compared using the formal TREC evaluation results to provide better insight into any changes observed (or not) in the clinical decision and timing results for the 2 systems.

There are a number of potentially confounding factors within the experimentation. A covariant analysis (repeated measure ANOVA) will be performed on the task number, task at total duration point (for fatigue), and time transitions (eg, 3-min task to 6-min task and 3-min task to 9-min task).

## Results

Recruiting began for the study in June 2018. As of April 4, 2019, there were 69 participants enrolled. The study is expected to close by May 30, 2019, with results to be published in July 2019.

## Discussion

The study is currently underway, and results will be reported at the conclusion of participant testing.

## Appendix

Multimedia Appendix 1 Pre-task questions and answer options.

Multimedia Appendix 2 Post tutorial instructions provided to the user prior to task assessment.

Multimedia Appendix 3 User interaction time capture . Taskiir user-interaction data capture detail for Stage two of the study, when the participant can use a search engine to help them to complete their task. Column 2 identifies all captured variables as either time-stamped events or calculated variables. Column 3 identifies where the event is triggered or how the variable is calculated.

Multimedia Appendix 4 Task Presentation Order : Latin square design of task presentation order. The presentation order is from left to right. The numbers in the table represent the task numbers. The subjects are denoted in the first column from S1 to S16. Two such squares (32 subjects) form a timing cohort. System selection is alternated for each column of the square starting with the State of Art system in column one. Note that the task order for the first 16 subjects are the same as for the second 16 subjects, however the search system used for each task is switched, i.e., so that the BM25 system is used for column 1 questions.

Multimedia Appendix 5 Task timing selection for each question. Timing cohorts of 32 people are identified in the top row as C1, C2 and C3. Each cohort will conduct the search for each task, as listed in the first column (T1, T2...T16), within the time constraint specified in minutes in the table.

## Abbreviations

ANOVA: analysis of variance
CDS: clinical decision support
DCG: discounted cumulative gain
IR: information retrieval
MAP: mean average precision
nDCG: normalized discounted cumulative gain
QREL: query relevance
RQ: research question
R-prec: R-precision
SERP: search engine results page
SML: scientific medical literature
TREC: text retrieval conference
UMLS: Unified Medical Language System
