# Supplementary material for: Impact of a Search Engine on Clinical Decisions Under Time and System Effectiveness Constraints: Research Protocol
Source: JMIR Res Protoc. 2019 May 28;8(5):e12803. doi: 10.2196/12803 (PMC6658292; doi:10.2196/12803)
Supplement: Multimedia Appendix 1 [file resprot_v8i5e12803_app1.pdf]

#	Question	Answer Option
1	Gender	(1) Male; (2) Female
2	Profession	(1) General Practitioner; (2) Specialist; (3) Intern, JHO, SHO, PHO or RMO; (4) Registrar; (5) Medical student; (6) Other - please specify
3	How many years of clinical experience do you have?	number
4	How would you rate your computer skills?	(1) Poor; (2) Fair; (3) Good; (4) Very good; (5) Excellent
5	How frequently do you use Medline or Pubmed for medical reference?	(1) never; (2) Once per month; (3) 2-3 times per month; (4) Once per week (5) More than once per week
6	How many hours of sleep did you have in the past 24 hours?	number
7	Is this amount of sleep average for you?	(1) It’s below average for me; (2) It’s average for me; (3) It’s above average for me
