# Supplementary figures and images for: Impact of a Search Engine on Clinical Decisions Under Time and System Effectiveness Constraints: Research Protocol
Source: JMIR Res Protoc. 2019 May 28;8(5):e12803. doi: 10.2196/12803 (PMC6658292; doi:10.2196/12803)

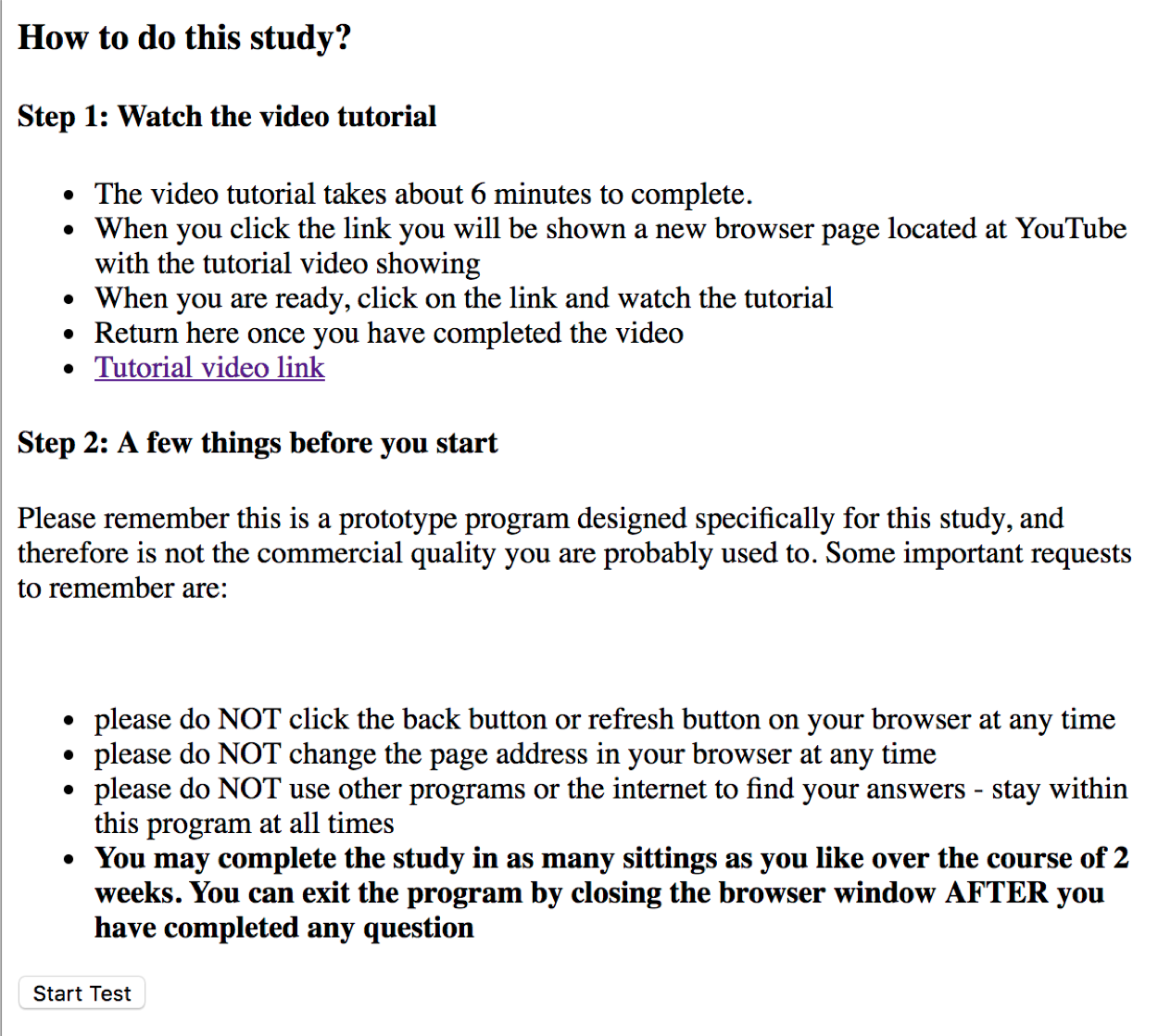

Supplement: Multimedia Appendix 2 [file resprot_v8i5e12803_app2.png]
