# Supplementary material for: Impact of a Search Engine on Clinical Decisions Under Time and System Effectiveness Constraints: Research Protocol
Source: JMIR Res Protoc. 2019 May 28;8(5):e12803. doi: 10.2196/12803 (PMC6658292; doi:10.2196/12803)
Supplement: Multimedia Appendix 3 [file resprot_v8i5e12803_app3.pdf]

**Id**	**Event/Variable (unit of measure)**	**Method of capture**
A.	Task_start_time (timestamp)	A timestamp is triggered when the search screen is presented to the user, together with the task
B.	Search_initiated (timestamp)	A timestamp is triggered when the user clicks on the 'Search' button or initiates the search with the keyboard
C.	Pre_search_dwell_interval (secs)	B-C
D.	SERP_reveal_time (timestamp)	A timestamp is triggered when the SERP has been revealed to the user
E.	System_search_interval (secs)	D-B
F.	User_SERP_click_time (timestamp)	A timestamp is triggered when the user clicks on any of the SERP snippets, in order to view the document
G.	SERP_review_interval (secs)	F-D
H.	Doc_revealed_time (timestamp)	A timestamp is triggered when the underlying document, related to the snippet the user clicked on the SERP, is revealed
I.	System_doc_reveal_interval (secs)	H-F
J.	User_saves_evidence (timestamp)	A timestamp is triggered when the user selects text from the document, which is saved to their evidence
K.	User_deletes_evidence (timestamp)	A timestamp is triggered when the user deletes an element of their saved evidence
L.	User_reports_relevance (timestamp)	A timestamp is triggered when the user selects the relevance for the document they are viewing
M.	User_finished_reading (timestamp)	A timestamp is triggered when the user closes the document review screen
N.	Document_read_interval (secs)	M-H
O.	User_movesto_completion (timestamp)	A timestamp is triggered when the user clicks on the button to complete the task
P.	User_completes_task (timestamp)	A timestamp is triggered when the user clicks on the complete task button and the fields are all validated
Q.	Answer_interval (secs)	P-O
R.	Task_completion_interval (secs)	P-A – (I+G)
