# Supplementary material for: Impact of a Search Engine on Clinical Decisions Under Time and System Effectiveness Constraints: Research Protocol
Source: JMIR Res Protoc. 2019 May 28;8(5):e12803. doi: 10.2196/12803 (PMC6658292; doi:10.2196/12803)
Supplement: Multimedia Appendix 4 [file resprot_v8i5e12803_app4.pdf]

Q order:	1	2	3	4	5	6	7	8	9	10	11	12	13	14	15	16
Subject																
S1	10	7	16	14	4	6	3	12	8	1	2	9	15	11	5	13
S2	11	8	1	15	5	7	4	13	9	2	3	10	16	12	6	14
S3	12	9	2	16	6	8	5	14	10	3	4	11	1	13	7	15
S4	13	10	3	1	7	9	6	15	11	4	5	12	2	14	8	16
S5	14	11	4	2	8	10	7	16	12	5	6	13	3	15	9	1
S6	15	12	5	3	9	11	8	1	13	6	7	14	4	16	10	2
S7	16	13	6	4	10	12	9	2	14	7	8	15	5	1	11	3
S8	1	14	7	5	11	13	10	3	15	8	9	16	6	2	12	4
S9	2	15	8	6	12	14	11	4	16	9	10	1	7	3	13	5
S10	3	16	9	7	13	15	12	5	1	10	11	2	8	4	14	6
S11	4	1	10	8	14	16	13	6	2	11	12	3	9	5	15	7
S12	5	2	11	9	15	1	14	7	3	12	13	4	10	6	16	8
S13	6	3	12	10	16	2	15	8	4	13	14	5	11	7	1	9
S14	7	4	13	11	1	4	3	9	5	14	15	6	12	8	2	10
S15	8	5	14	12	2	5	4	10	6	15	16	7	13	9	3	11
S16	9	6	15	13	3	6	5	11	7	16	1	8	14	10	4	12
