# Supplementary material for: Impact of a Search Engine on Clinical Decisions Under Time and System Effectiveness Constraints: Research Protocol
Source: JMIR Res Protoc. 2019 May 28;8(5):e12803. doi: 10.2196/12803 (PMC6658292; doi:10.2196/12803)
Supplement: Multimedia Appendix 5 [file resprot_v8i5e12803_app5.pdf]

Timing Cohort:	C1	C2	C3
Task Number			
T1	3	6	9
T2	3	9	6
T3	6	3	9
T4	6	9	3
T5	9	3	6
T6	9	6	3
T7	3	6	9
T8	3	9	6
T9	6	3	9
T10	6	9	3
T11	9	3	6
T12	9	6	3
T13	3	6	9
T14	9	6	3
T15	6	3	9
T16	6	9	3
Total Mins:	96	96	96
